# Supplementary material for: Human degradation of tropical moist forests is greater than previously estimated
Source: Nature. 2024 Jul 3;631(8021):570–6. doi: 10.1038/s41586-024-07629-0 (PMC11254752; doi:10.1038/s41586-024-07629-0)
Supplement: Supplementary file 1 — Supplementary Table and figures supporting the results and methodology [file 41586_2024_7629_MOESM1_ESM.pdf]

nature portfolio

https://doi.org/10.1038/s41586-024-07629-0

Supplementary information

Human degradation of tropical moist forests is greater than previously estimated

In the format provided by the authors and unedited

Supplementary Table.1 Number of GEDI shots and statistics (mean, standard deviation and precision) of grid cells by continent of AGBD prediction standard error (AGBD_SE) for intact forest. The precision of the AGBD_SE was obtained by computing the standard error of the AGBD_SE values at footprint level for each continent.

**Continent**	**Number of GEDI shots**	**Mean AGBD_SE [Mg/ha]**	**Standard Deviation [Mg/ha]**	**Precision of the Mean AGBD_SE [Mg/ha]**
Africa	8986956	17	0.0043875	1.464E-06
Americas	25303980	12.99978	0.0265894	5.286E-06
Asia	2519005	12.49522	4.476483	0.0028205

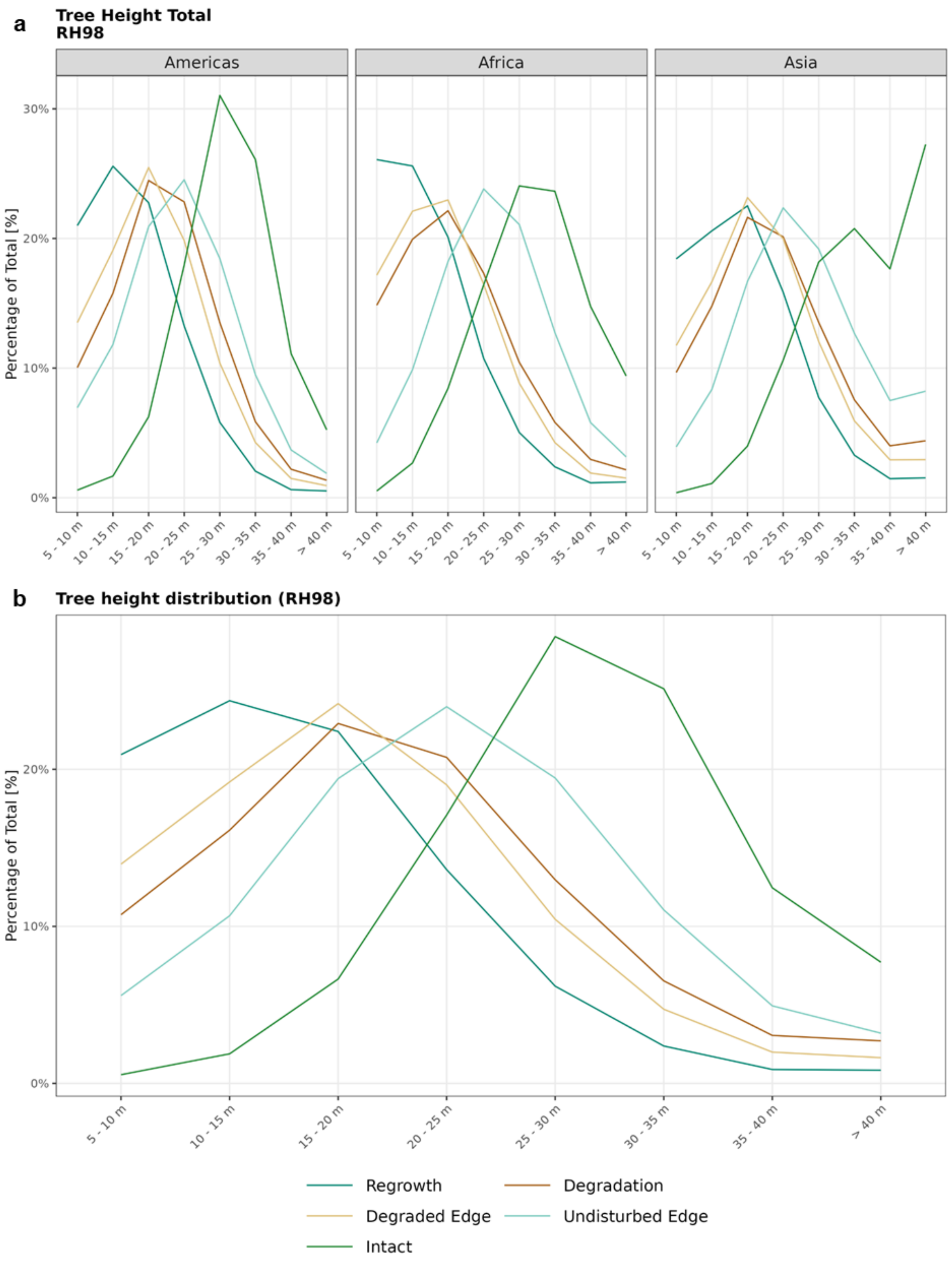

Supplementary Fig.1 Distribution of RH98 for intact forests, degraded forests, undisturbed forest edges, degraded forest edges and forest regrowths at the continental (panel a) and pantropical level (panel b).

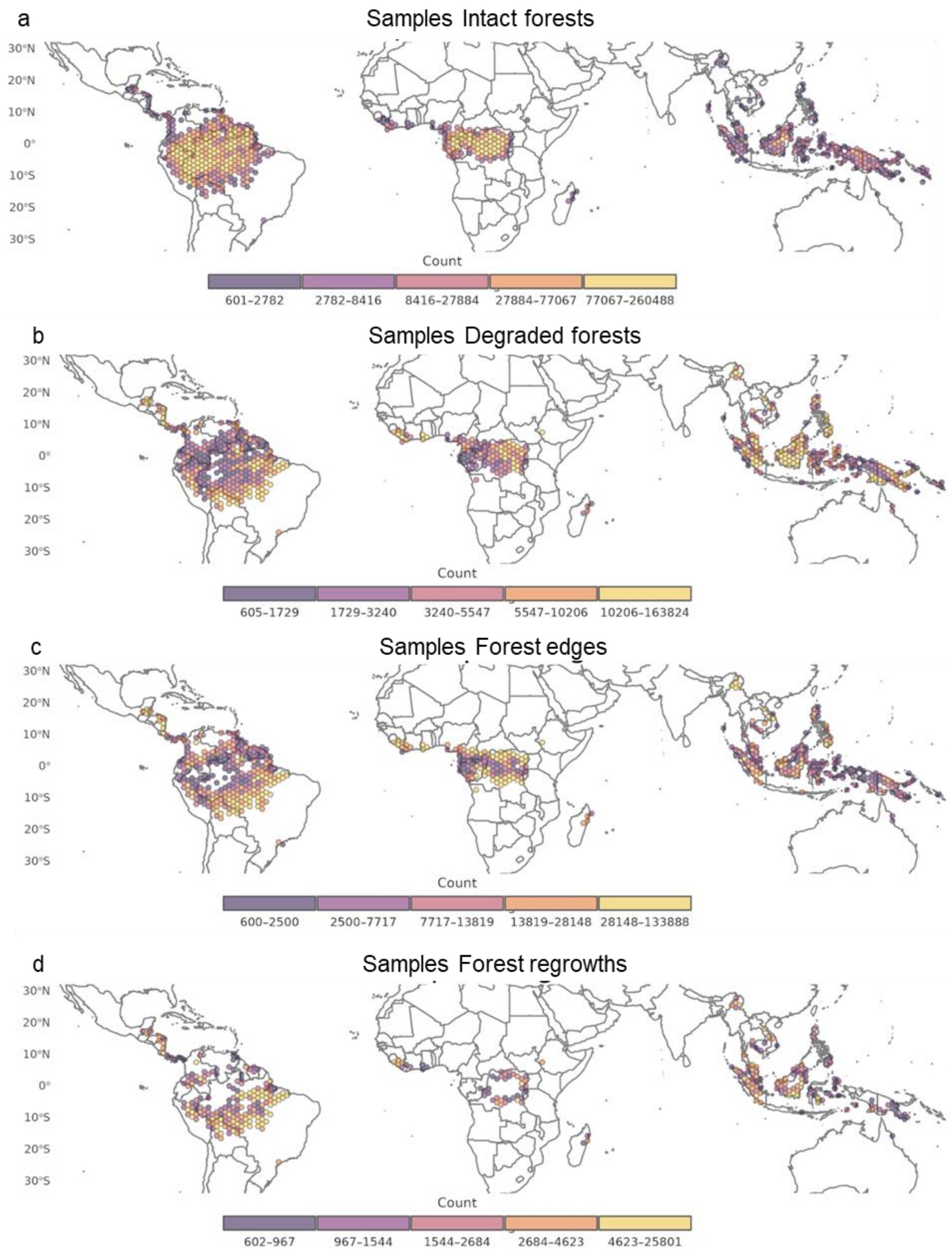

Supplementary Fig.2 Number of GEDI samples for intact forests (a), degraded forests from logging, fire or natural disturbances (b); forest/non-forest edges (120 m width) (c); and forest regrowths (d); in 1.5° (side ~ 167 km) hexagon grid cells between 30°N and 30°S.

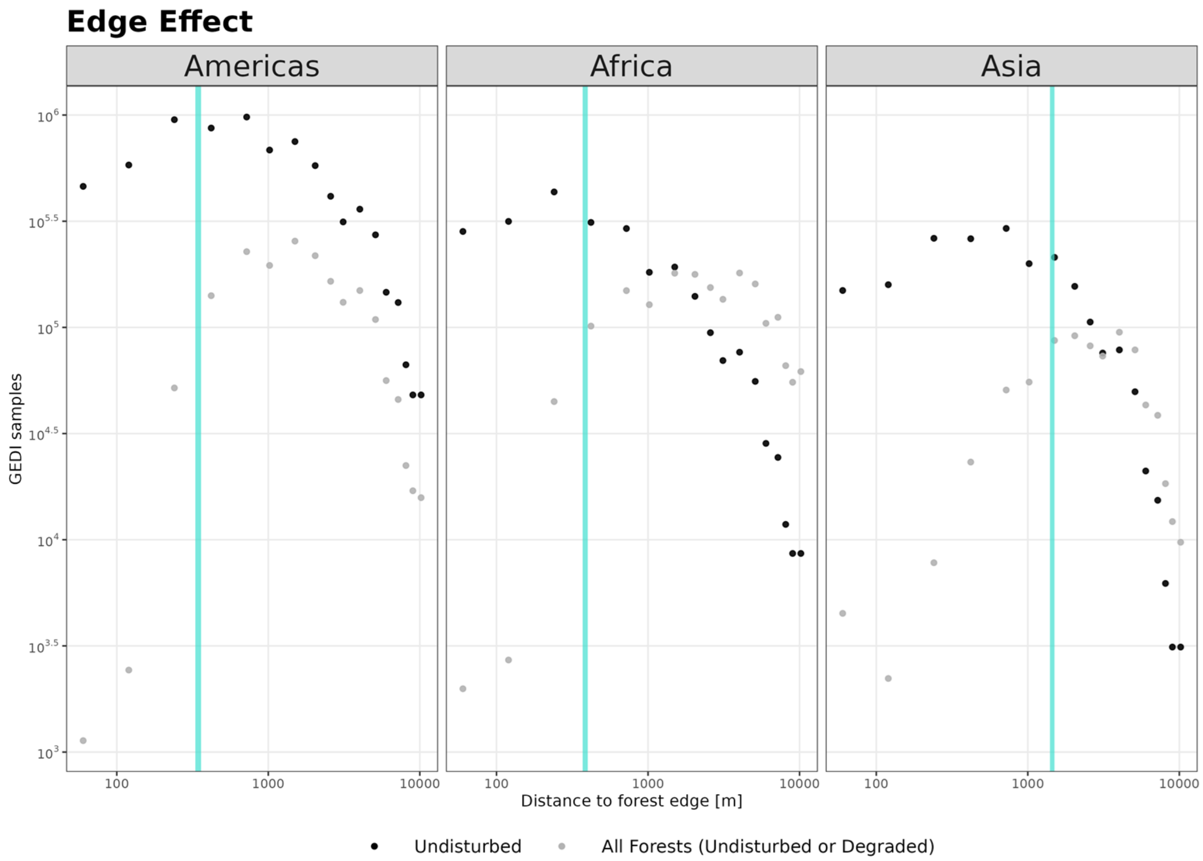

Supplementary Fig.3 Number of GEDI samples for undisturbed and all forests (including undisturbed and degraded forest) at various distances to the forest edge (from 60 m to 10200 m).

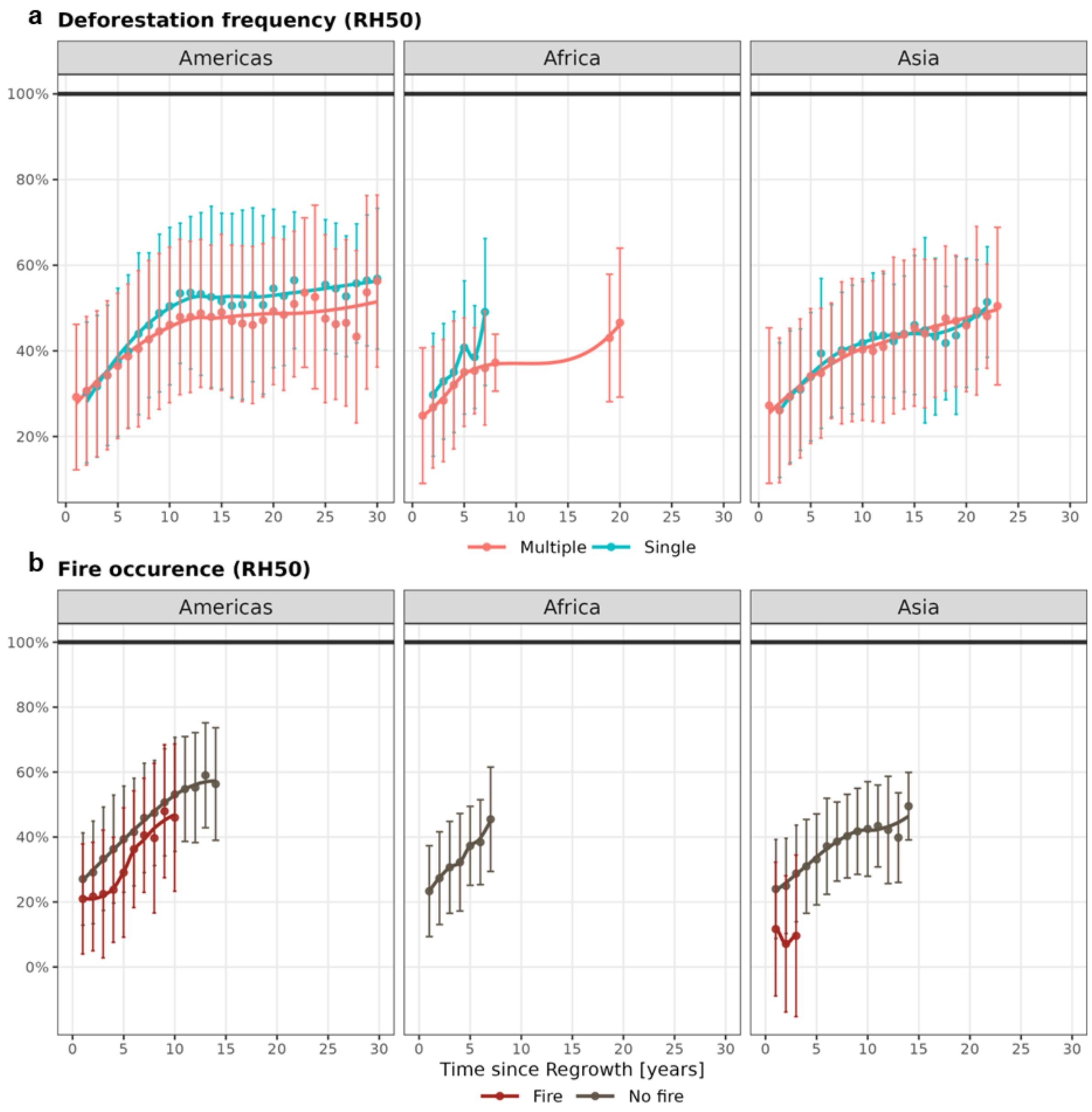
a
Deforestation frequency (RH50)
Americas
Africa
Asia
100%
80%
60%
40%
20%
0
5
10
15
20
25
30
Multiple
Single
b
Fire occurence (RH50)
Americas
Africa
Asia
100%
80%
60%
40%
20%
0%
Time since Regrowth [years]
Fire
No fire

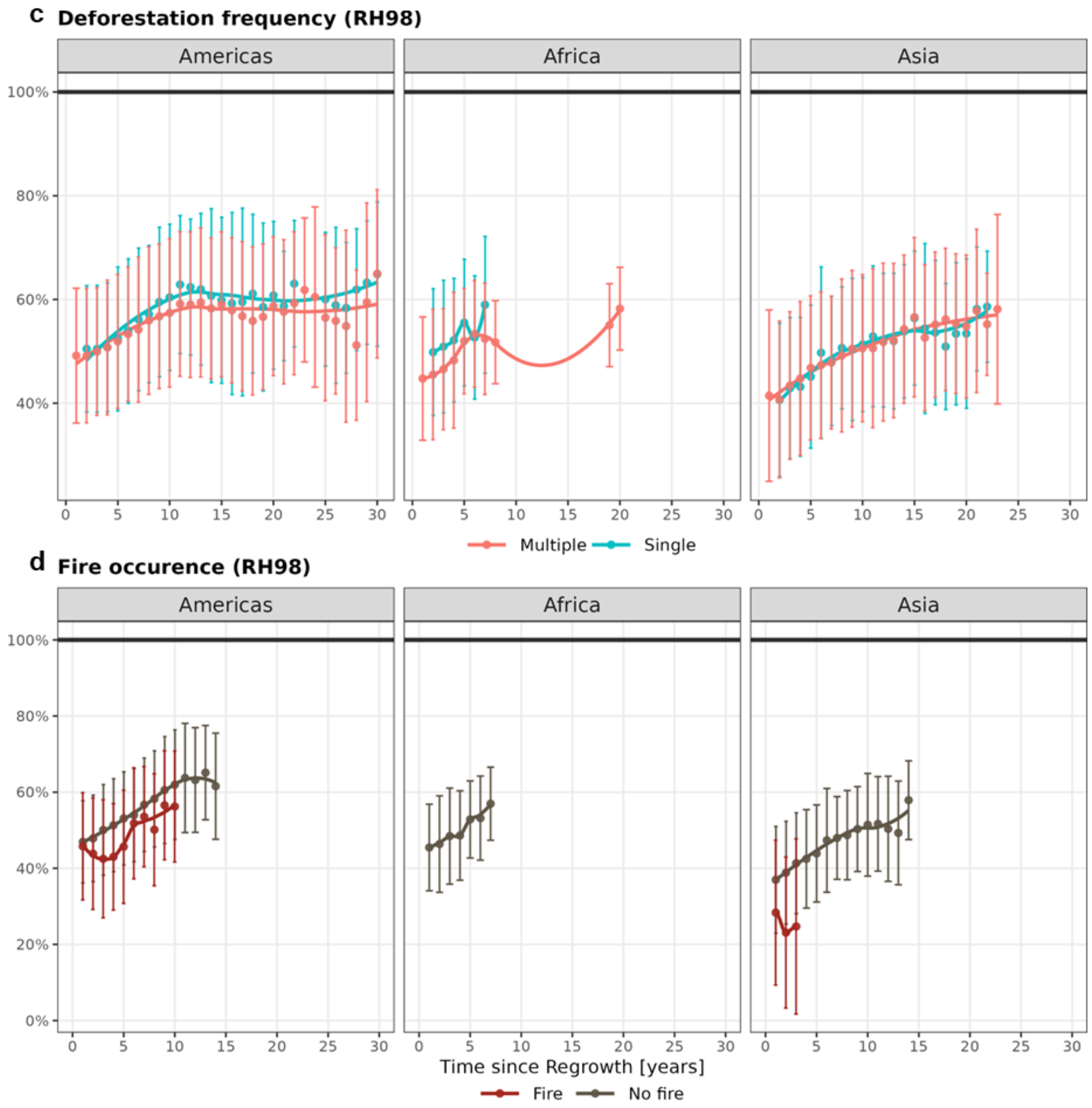
c Deforestation frequency (RH98)
Americas
Africa
Asia
100%
80%
60%
40%
0
5
10
15
20
25
30
Multiple
Single
d Fire occurence (RH98)
Americas
Africa
Asia
100%
80%
60%
40%
20%
0%
Time since Regrowth [years]
Fire
No fire

e Deforestation frequency

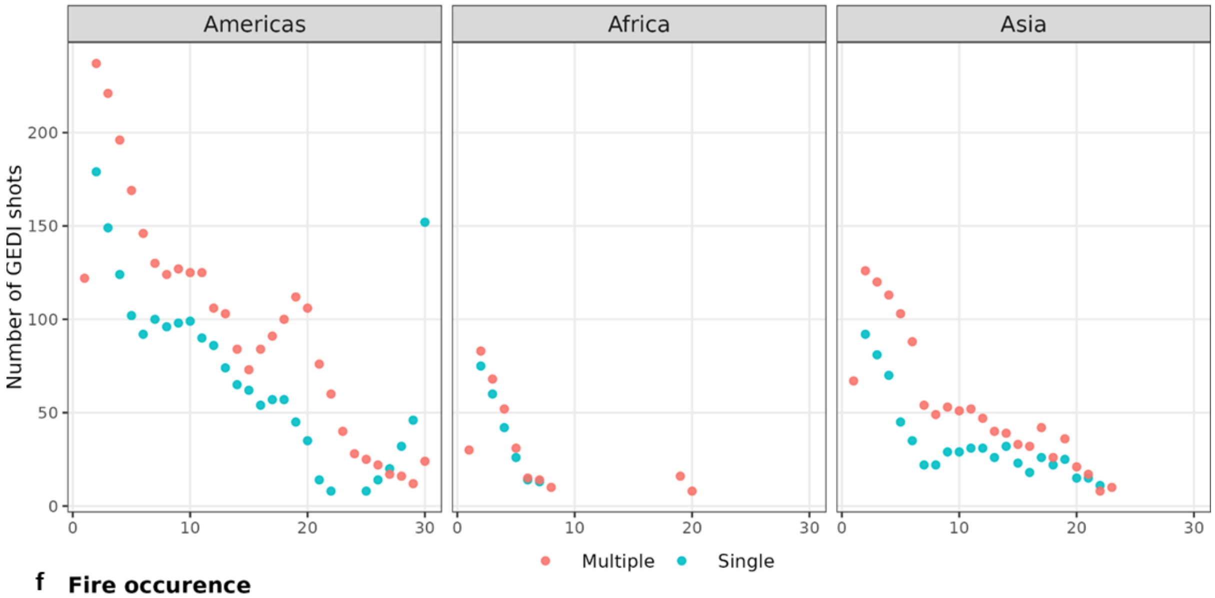

f Fire occurence

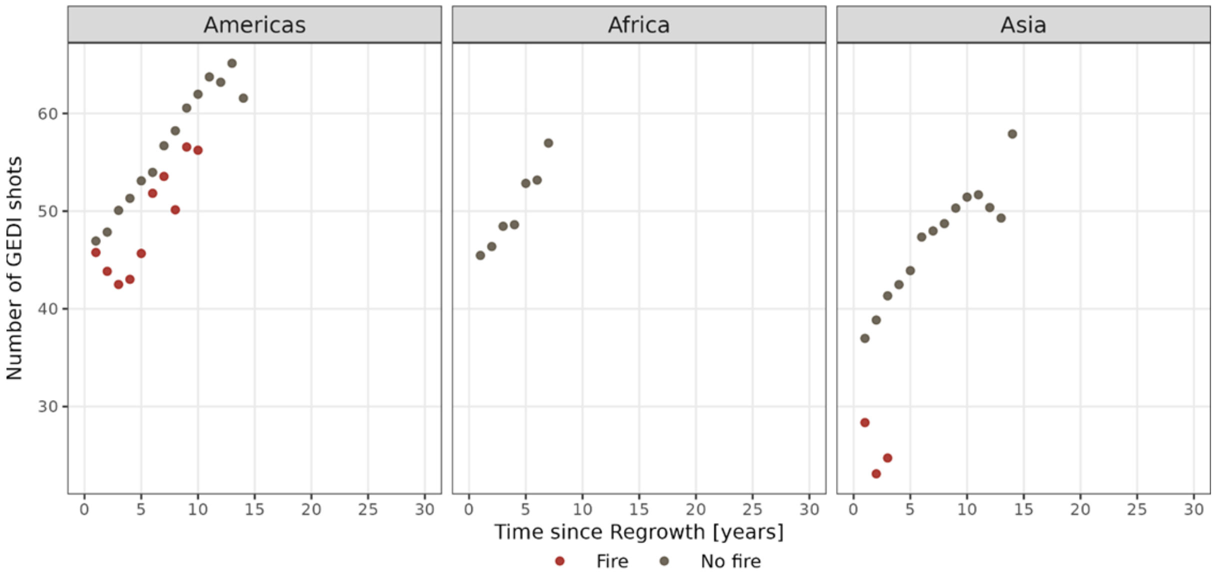

Supplementary Fig.4 Long-term recovery of relative heights (RH50 and RH98) of secondary forests regrowing on abandoned deforested lands. Previous land use type and intensity are assessed on the basis of two proxies: (panels a and c) Forest regrowing after single or multiple deforestation events (repeated cycles events of minimum 3 years duration) and (panels b,and d) forest regrowing after forest fires or not (identified in the GFC loss from forest product from UMD GLAD 2001-2021). Results are reported as the percentage of intact forest relative heights (solid line) after normalising the difference in height within each grid cell between intact forest and each forest regrowth type and age. The solid lines depict the local polynomial regression (loess) fit to the data. Dots represent the average value of RH50/RH98 and vertical bars indicate the spatial standard deviation. Panel e and f show the associated number of GEDI samples for the two proxies.

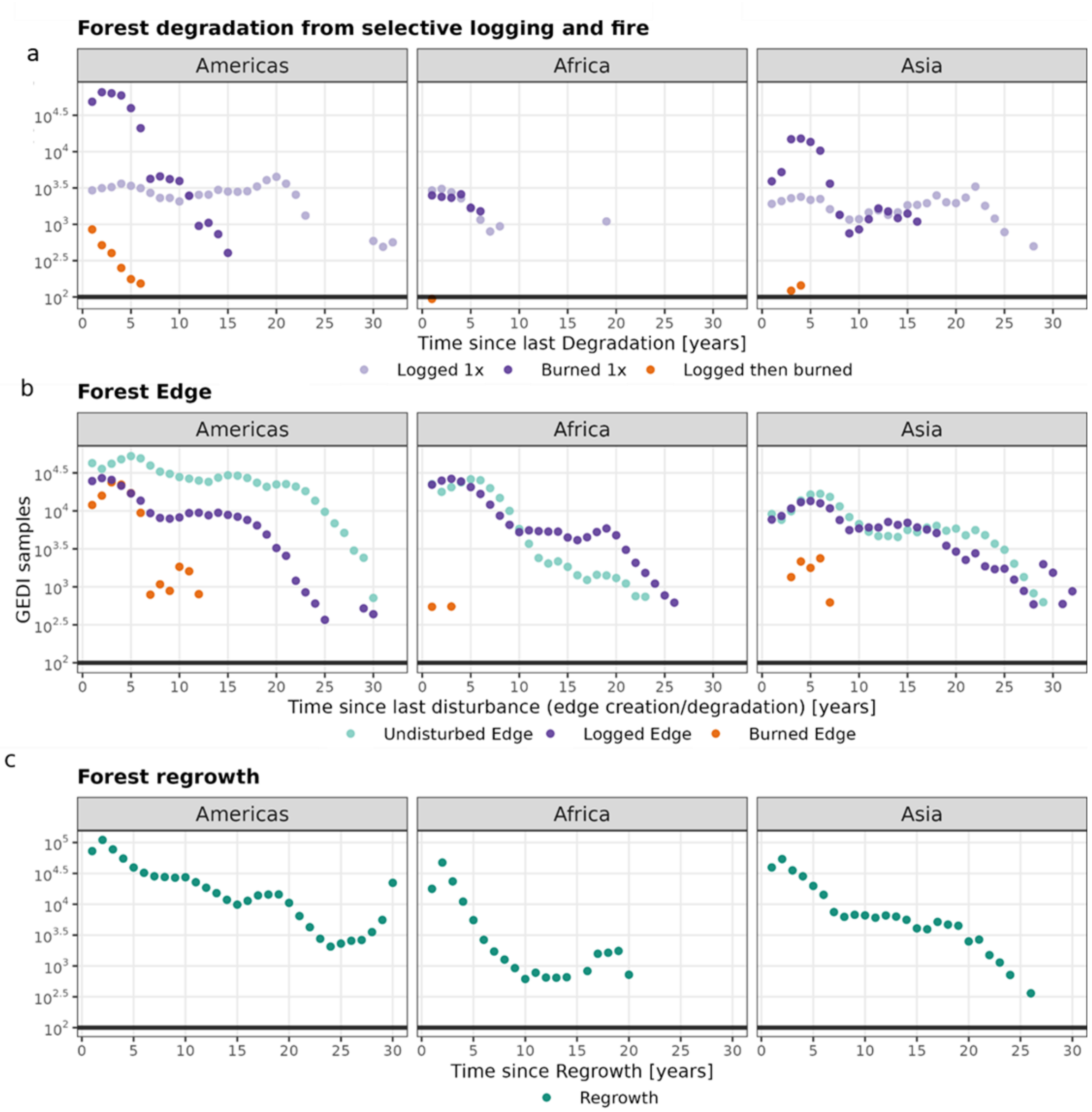

Supplementary Fig.5 Number of GEDI samples for degraded forests from selective logging and/or fire (a); for forest edges that remain undisturbed, or suffer from logging or fire (b) and for forest regrowths (c).

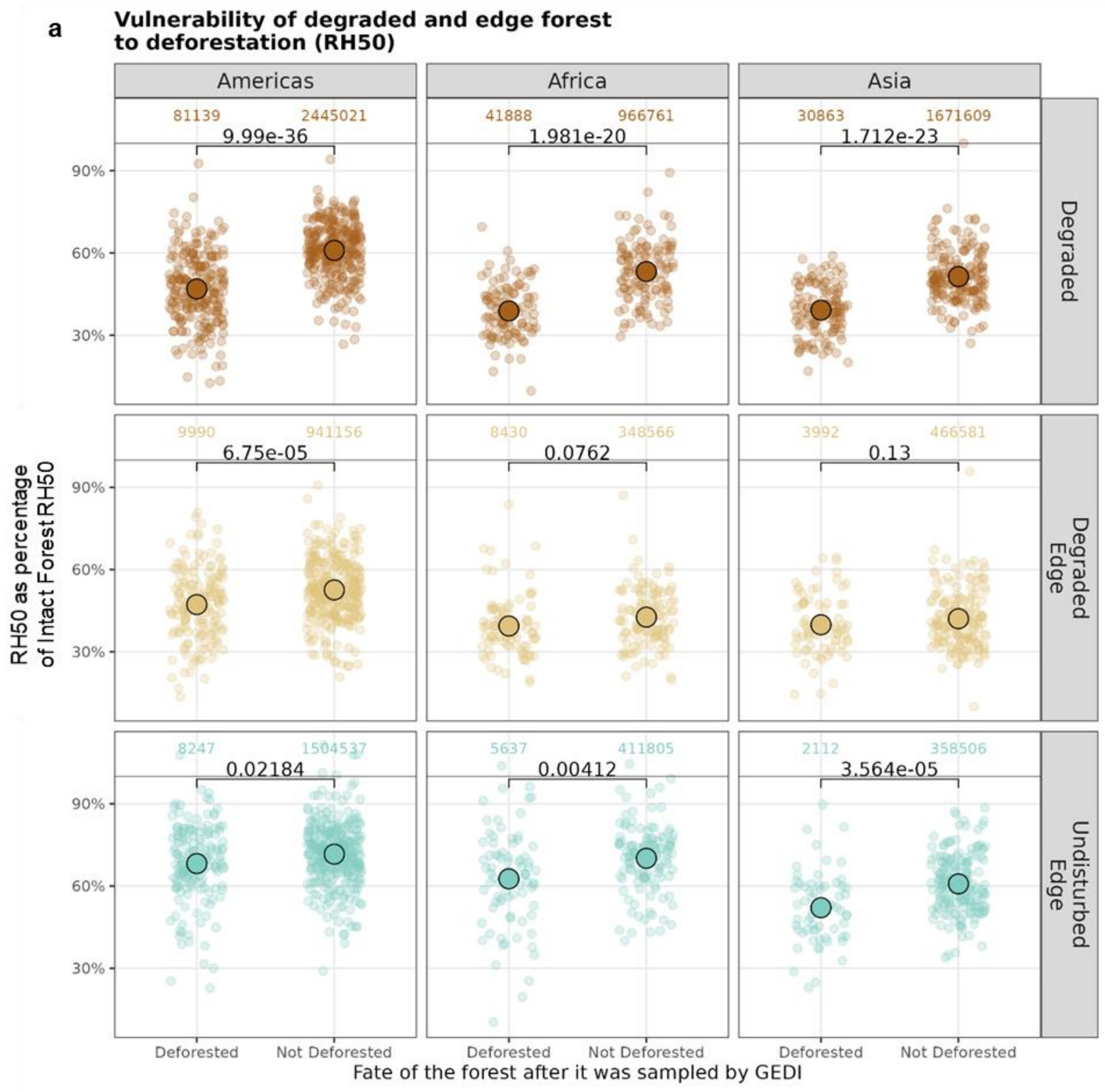
a
Vulnerability of degraded and edge forest to deforestation (RH50)
Americas
Africa
Asia
81139
2445021
9.99e-36
41888
966761
1.981e-20
30863
1671609
1.712e-23
Degraded
9990
941156
6.75e-05
8430
348566
0.0762
3992
466581
0.13
Degraded Edge
8247
1504537
0.02184
5637
411805
0.00412
2112
358506
3.564e-05
Undisturbed Edge
90%
60%
30%
RH50 as percentage of Intact Forest RH50
Deforested
Not Deforested
Fate of the forest after it was sampled by GEDI

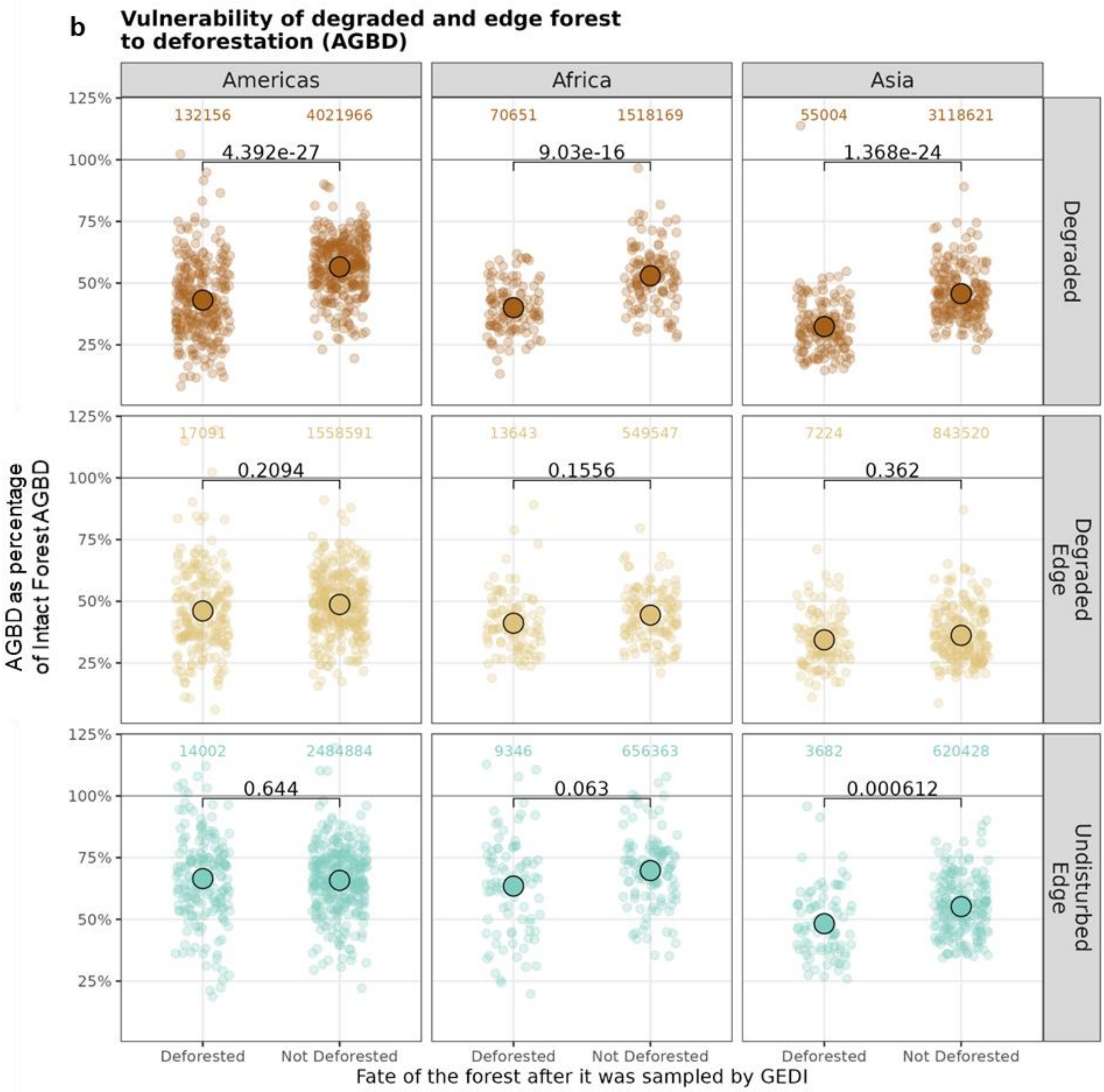

b
Vulnerability of degraded and edge forest
to deforestation (AGBD)
Americas
Africa
Asia
Degraded
Degraded Edge
Undisturbed Edge
132156
4021966
4.392e-27
70651
1518169
9.03e-16
55004
3118621
1.368e-24
17091
1558591
0.2094
13643
549547
0.1556
7224
843520
0.362
14002
2484884
0.644
9346
656363
0.063
3682
620428
0.000612
125%
100%
75%
50%
25%
AGBD as percentage of Intact Forest AGBD
Deforested
Not Deforested
Fate of the forest after it was sampled by GEDI

Supplementary Fig.6 Differences in RH50 (panel a) and AGBD (panel b) of different forest types prior to deforestation, compared to RH50 and AGBD of similar and contemporary forests that are not deforested. The RH50 and AGBD are all retrieved during the period 2019-2021, while the deforestation events occurred 1-3 years after GEDI measurements (2020-2022). The different forest types are degraded forest (located beyond the edge), degraded edge forest (width 120 m), and undisturbed edge forests. For degraded forests, degradation occurred before 2019, and no disturbance was observed during the year of GEDI data acquisition. Big circles represent the averages, and the small dots are individual GEDI samples. Adjusted p values are determined by two-tailed unpaired T test. The number at the top of each distribution corresponds to the number of GEDI samples.

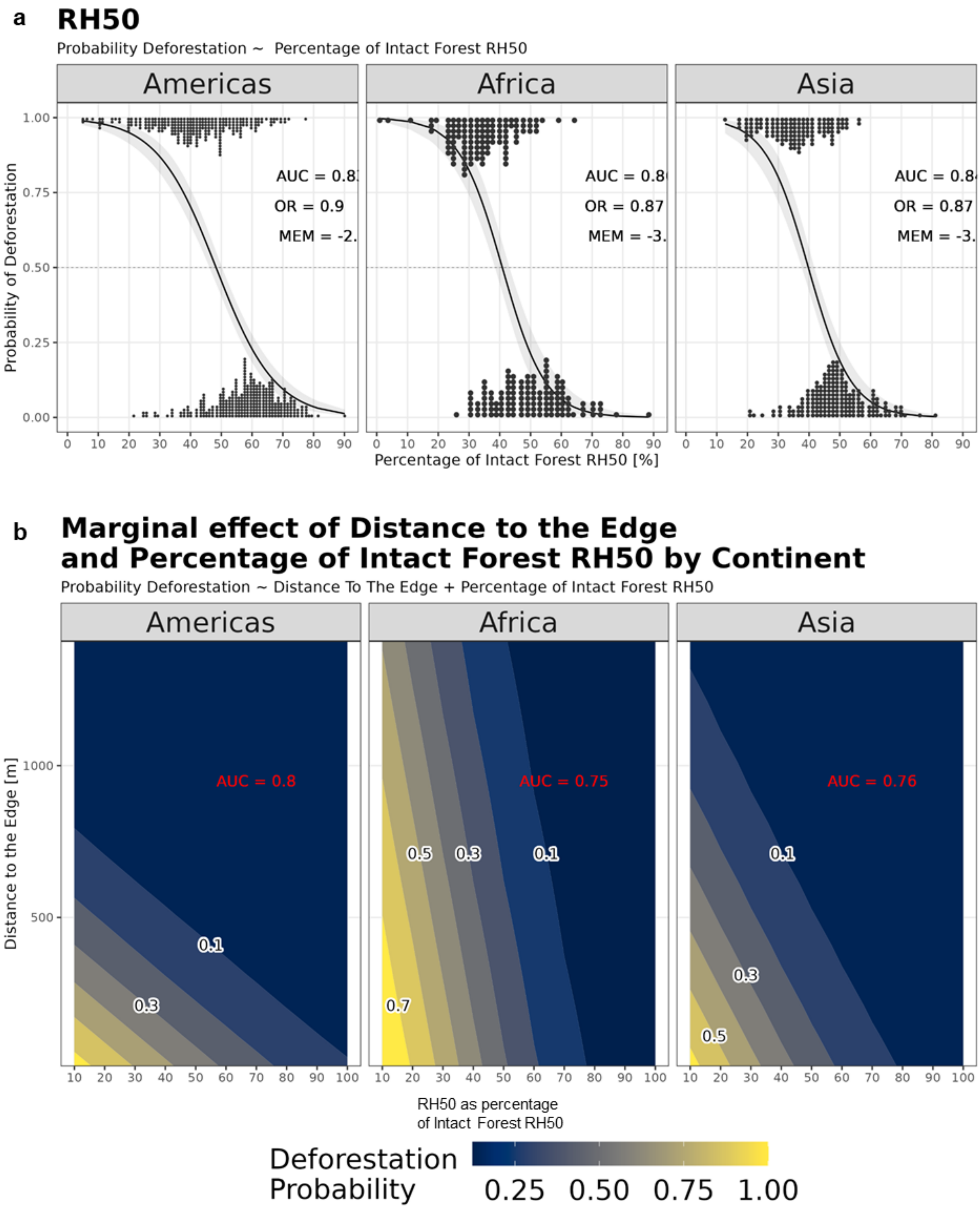
a
RH50
Probability Deforestation ~ Percentage of Intact Forest RH50
Americas
Africa
Asia
AUC = 0.8
OR = 0.9
MEM = -2.
AUC = 0.8
OR = 0.87
MEM = -3.
AUC = 0.8
OR = 0.87
MEM = -3.
Probability of Deforestation
Percentage of Intact Forest RH50 [%]
b
Marginal effect of Distance to the Edge
and Percentage of Intact Forest RH50 by Continent
Probability Deforestation ~ Distance To The Edge + Percentage of Intact Forest RH50
Americas
Africa
Asia
AUC = 0.8
AUC = 0.75
AUC = 0.76
Distance to the Edge [m]
RH50 as percentage
of Intact Forest RH50
Deforestation
Probability
0.25 0.50 0.75 1.00

Supplementary Fig.7 a) Marginal model plot of the binary logistic regression model of RH50 predicting whether deforestation was reported. The plot shows the expected influence of degraded forest structure (expressed as a percentage of intact forest RH50) on the probability of deforestation. The grey shaded areas indicate the 95% credibility intervals of the predicted values. OR is the odds ratio. Stacked dots represent the GEDI samples deforested/not deforested. The marginal effect at the mean (MEM) quantifies for a one-point increase in RH50 (i.e. the x-axis) the associated percentage point variation in the probability of deforestation. AUC is the area under the ROC curve. Marginal effects are partial derivatives of the regression equation for each variable in the model for each unit in the data. b) Plot of the marginal effects showing the probability of deforestation based on degraded forest structure (expressed as a percentage of intact forest RH50) and the distance to the forest edge.

a

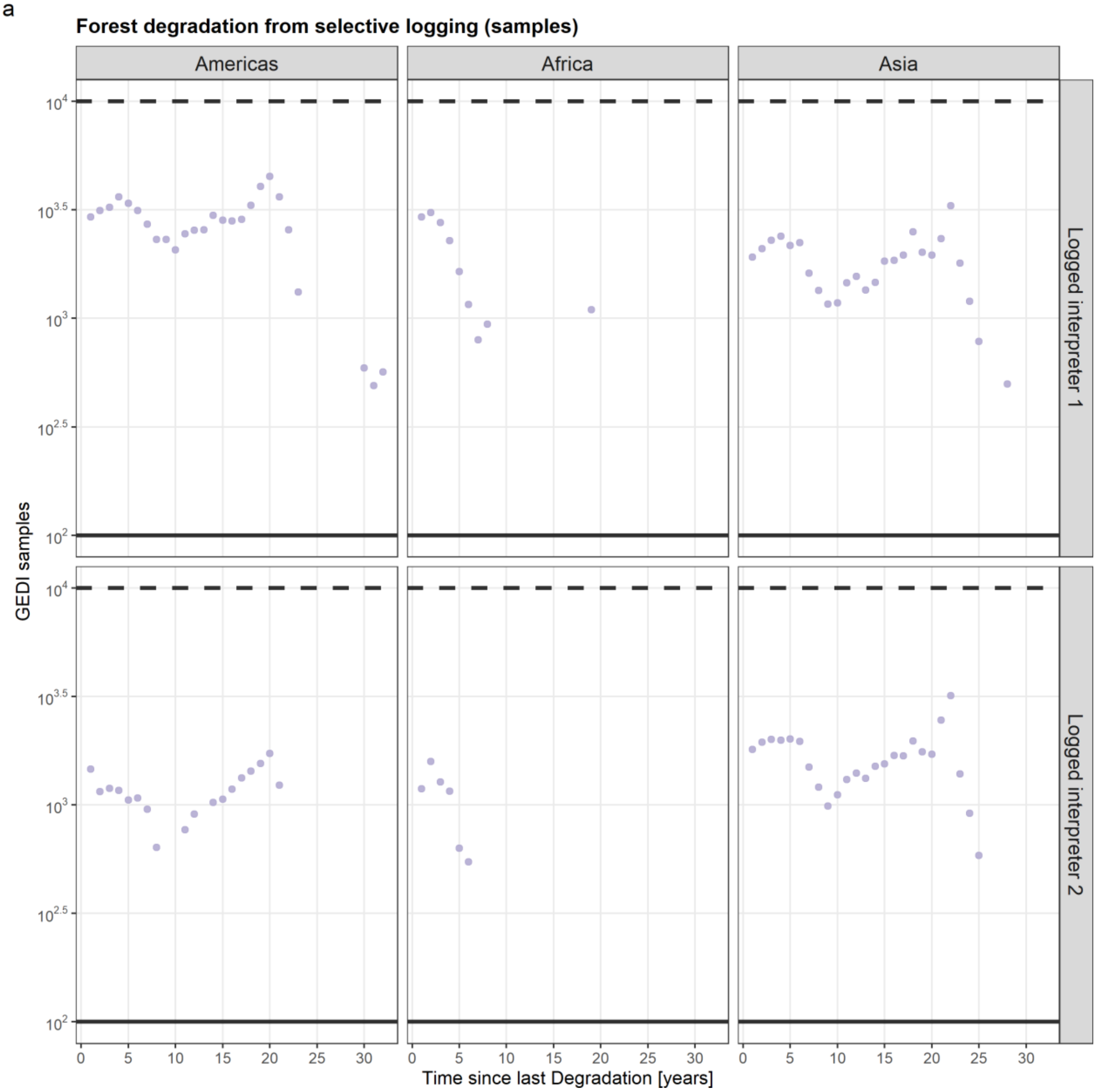

Forest degradation from selective logging (samples)
Americas
Africa
Asia
Logged interpreter 1
Logged interpreter 2
GEDI samples
Time since last Degradation [years]

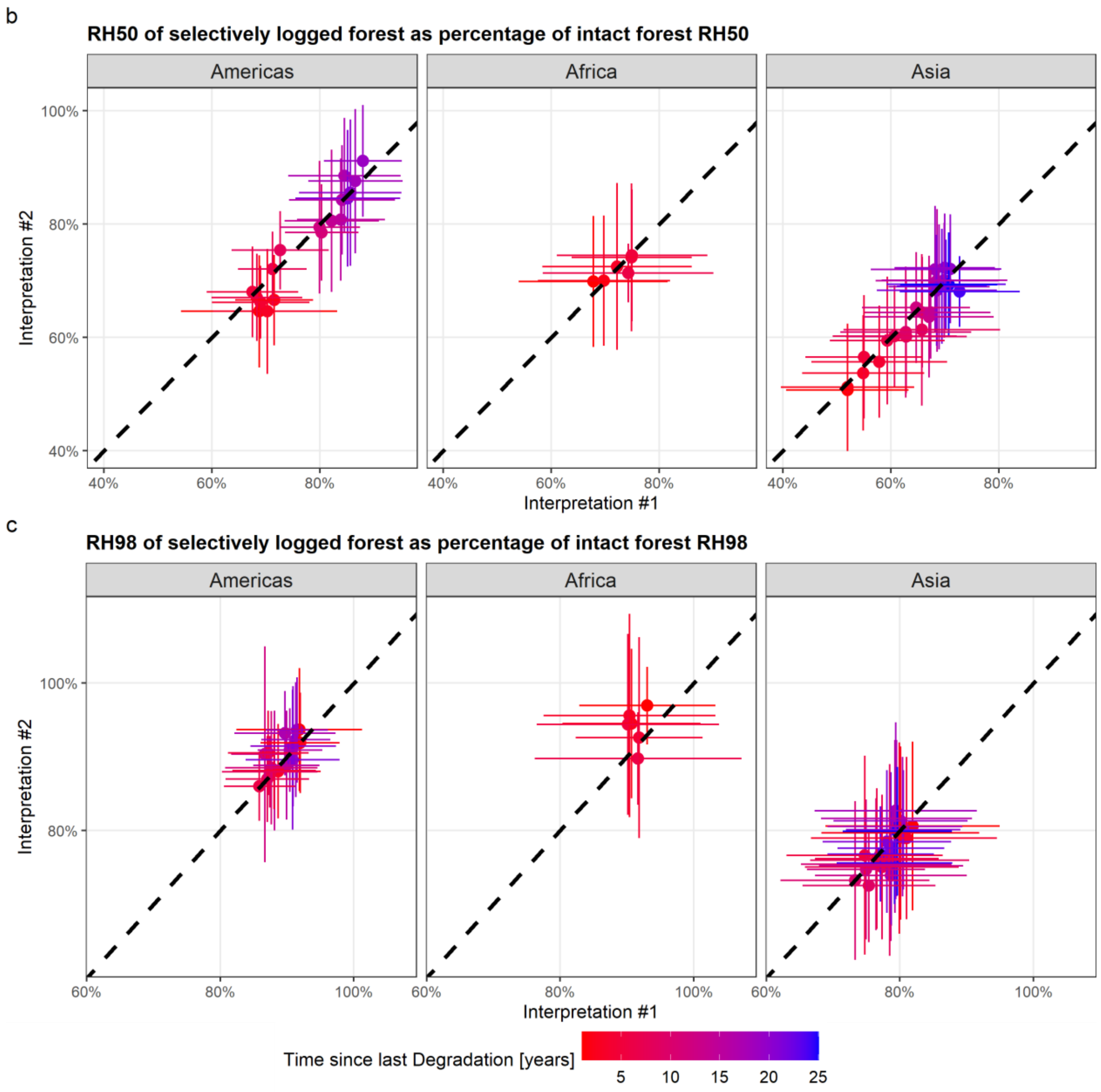

Supplementary Fig.8 Comparative analysis of the impact of selective logging (with timing attribution to the JRC TMF dataset) between the use of manual delineation performed by interpreter 1 (corresponding to the data collection of the manuscript) vs the delineation performed by interpreter 2 (sensitivity analysis). Panels a to c refer to the number of sampled GEDI footprints, impacts of logging on RH50 and RH98 respectively for each continent and attribution scheme. In panel b and c, horizontal and vertical bars indicate the spatial standard deviation following the delineation of selective logging made by interpretation #1 and #2 respectively. The dashed line in panel b and c is the 1:1 line.

a

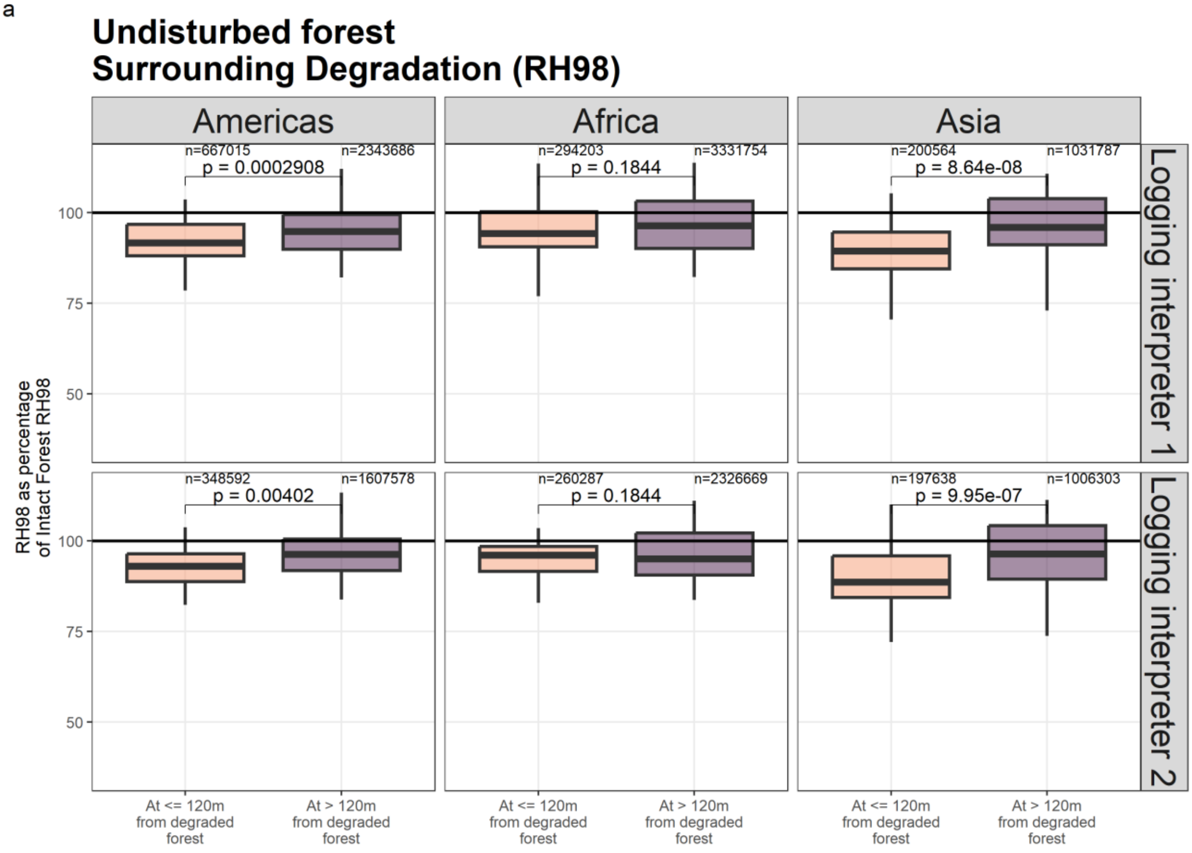
Undisturbed forest
Surrounding Degradation (RH98)
Americas
Africa
Asia
Logging interpreter 1
Logging interpreter 2
RH98 as percentage of Intact Forest RH98
n=667015
n=2343686
p = 0.0002908
n=294203
n=3331754
p = 0.1844
n=200564
n=1031787
p = 8.64e-08
n=348592
n=1607578
p = 0.00402
n=260287
n=2326669
p = 0.1844
n=197638
n=1006303
p = 9.95e-07
100
75
50
At <= 120m from degraded forest
At > 120m from degraded forest

b

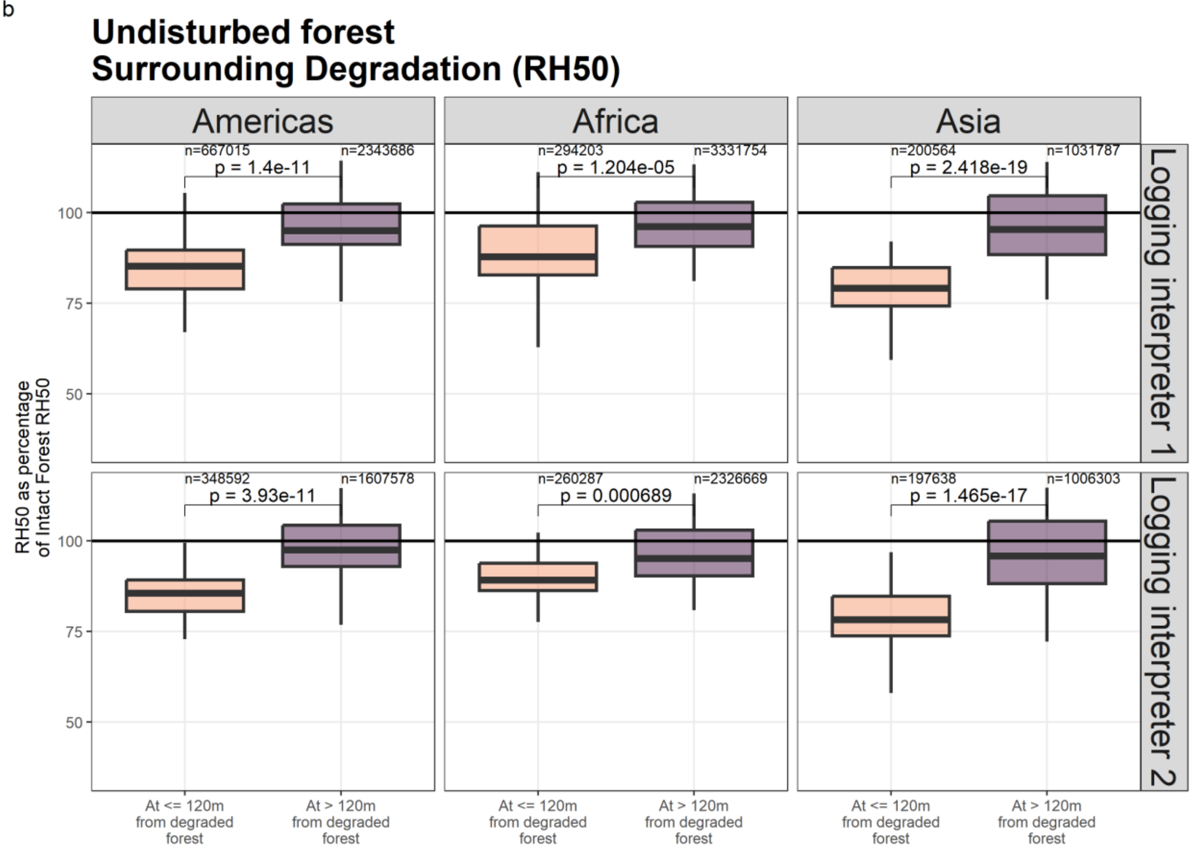
Undisturbed forest
Surrounding Degradation (RH50)
Americas
Africa
Asia
Logging interpreter 1
Logging interpreter 2
RH50 as percentage of Intact Forest RH50
n=667015
n=2343686
p = 1.4e-11
n=294203
n=3331754
p = 1.204e-05
n=200564
n=1031787
p = 2.418e-19
n=348592
n=1607578
p = 3.93e-11
n=260287
n=2326669
p = 0.000689
n=197638
n=1006303
p = 1.465e-17
100
75
50
At <= 120m from degraded forest
At > 120m from degraded forest

Supplementary Fig.9 Comparative analysis between the difference in RH98 (panel a) and RH50 (panel b) for forest classified as undisturbed in the JRC-TMF dataset located within and outside a buffer area (120 m radius) around logged forest delineated by interpreter 1 (corresponding to the data collection of the manuscript) vs interpreter 2 (sensitivity analysis). Adjusted p values are determined by two-tailed unpaired T test. The number on top of each boxplot corresponds to the number of GEDI samples. Boxplot shows data from the 25th–75th percentile, the median (line) and whiskers extending to the minimum and maximum within 1.5× interquartile range.

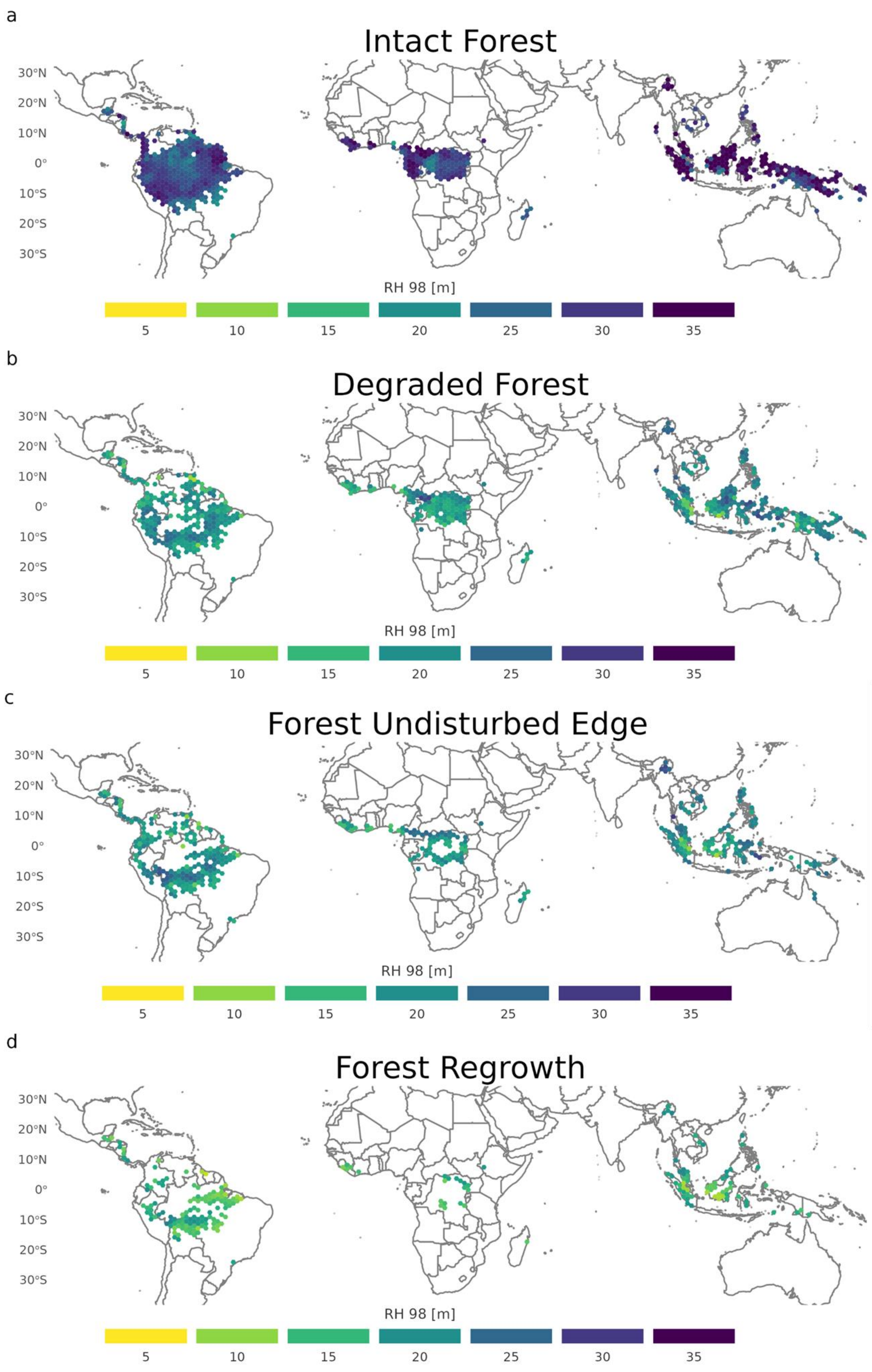
a
Intact Forest
30°N
20°N
10°N
0°
10°S
20°S
30°S
RH 98 [m]
5
10
15
20
25
30
35
b
Degraded Forest
30°N
20°N
10°N
0°
10°S
20°S
30°S
RH 98 [m]
5
10
15
20
25
30
35
c
Forest Undisturbed Edge
30°N
20°N
10°N
0°
10°S
20°S
30°S
RH 98 [m]
5
10
15
20
25
30
35
d
Forest Regrowth
30°N
20°N
10°N
0°
10°S
20°S
30°S
RH 98 [m]
5
10
15
20
25
30
35

Supplementary Fig.10 Random sampling procedure result showing the variability of canopy height distribution (RH98) for intact (a), degraded (b), edge forests (c) and forest regrowth (d). The random sampling procedure consists of sampling 300 GEDI observations for each grid cell, iterating 500 times this sampling and computing the average values. Vertical bars indicate the standard deviation.

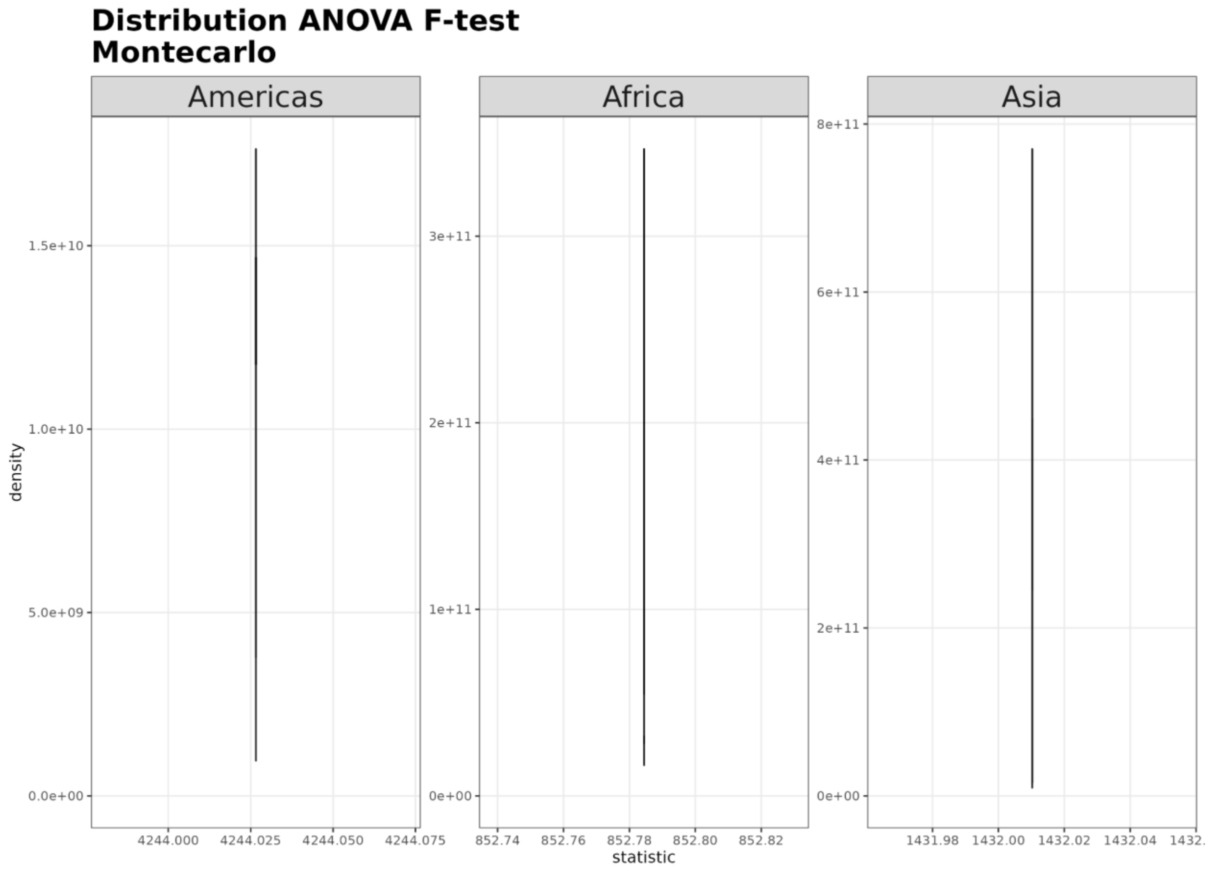

Supplementary Fig.11 Distribution of Anova f-test after Monte Carlo simulation to propagate AGBD prediction standard error in the analysis of edge effects.

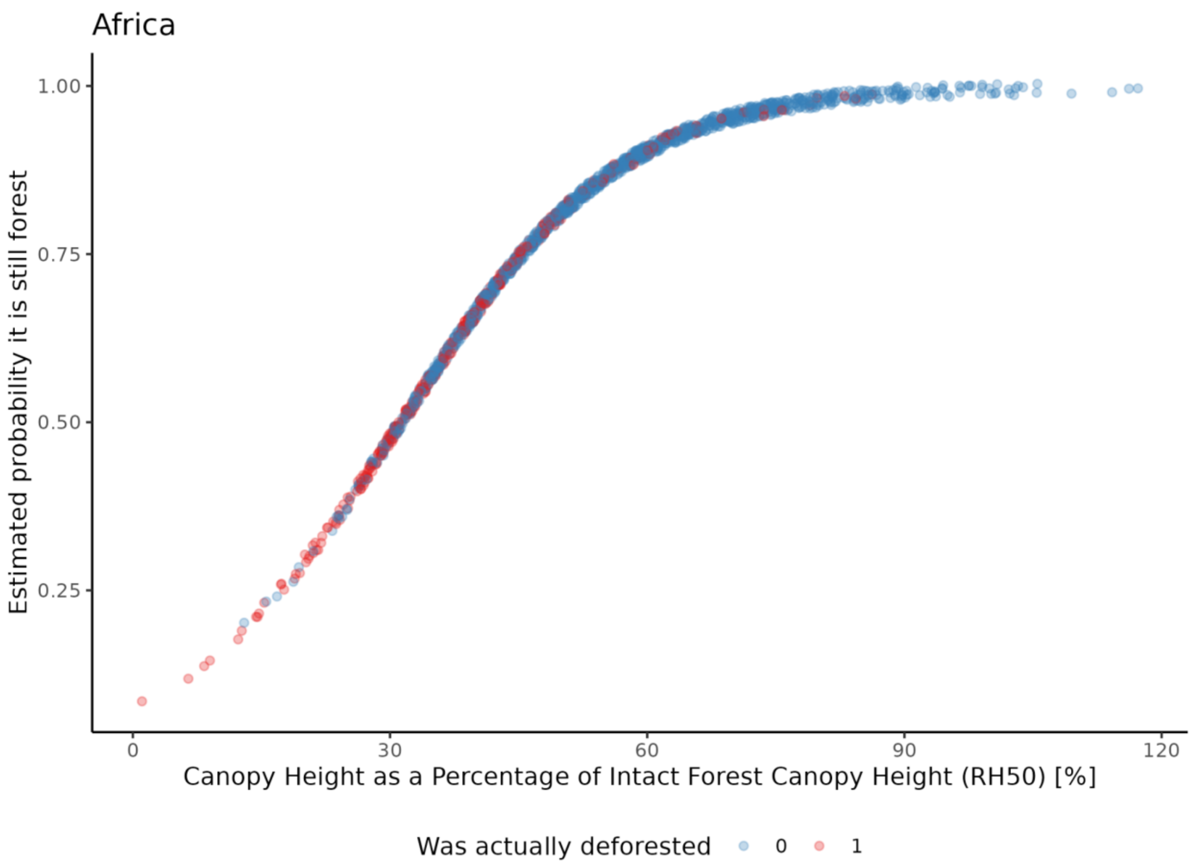

Supplementary Fig.12 Logistic regression probability with RH50 data over Africa. The logistic curve shows whether a forest is deforested or not (red and blue colours, respectively) based on the RH50 metric (expressed as a percentage of Intact forest RH50).

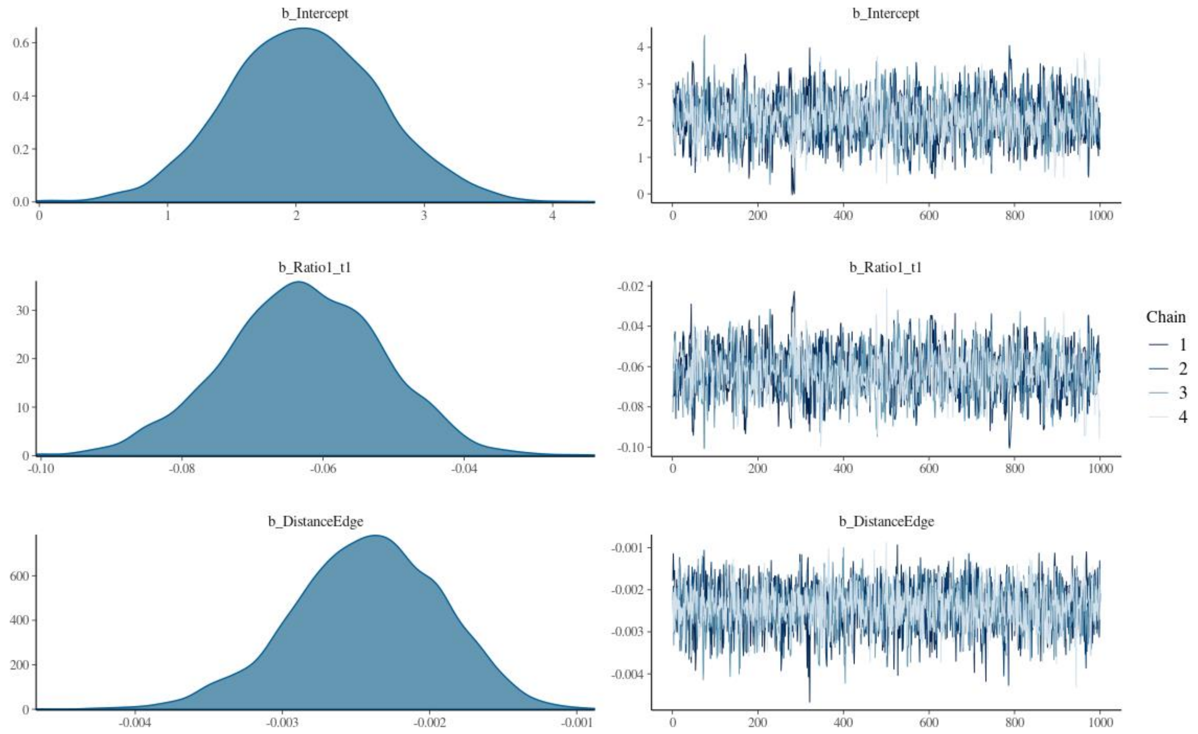

Supplementary Fig.13 Posterior distributions and trace plots for the parameters of the binomial GLM models fitted within a Bayesian framework. Right: Trace plots showing the four chains. *Ratio1_t1* is the percentage of intact forest canopy height (RH98) and *DistanceEdge* is the distance to the edge. A visual inspection of the trace plots indicates good mixing among the 4 chains.
