# Supplementary material for: Human degradation of tropical moist forests is greater than previously estimated
Source: Nature. 2024 Jul 3;631(8021):570–6. doi: 10.1038/s41586-024-07629-0 (PMC11254752; doi:10.1038/s41586-024-07629-0)
Supplement: Supplementary file 2 — Reporting Summary [file 41586_2024_7629_MOESM2_ESM.pdf]

nature portfolio

Corresponding author(s): Clement Bourgoin

Last updated by author(s): Apr 30, 2024

Reporting Summary

Nature Portfolio wishes to improve the reproducibility of the work that we publish. This form provides structure for consistency and transparency in reporting. For further information on Nature Portfolio policies, see our Editorial Policies and the Editorial Policy Checklist.

Statistics

For all statistical analyses, confirm that the following items are present in the figure legend, table legend, main text, or Methods section.

n/a	Confirmed	
☐	☒	The exact sample size (*n*) for each experimental group/condition, given as a discrete number and unit of measurement
☐	☒	A statement on whether measurements were taken from distinct samples or whether the same sample was measured repeatedly
☐	☒	The statistical test(s) used AND whether they are one- or two-sided<br>*Only common tests should be described solely by name; describe more complex techniques in the Methods section.*
☒	☐	A description of all covariates tested
☒	☐	A description of any assumptions or corrections, such as tests of normality and adjustment for multiple comparisons
☐	☒	A full description of the statistical parameters including central tendency (e.g. means) or other basic estimates (e.g. regression coefficient) AND variation (e.g. standard deviation) or associated estimates of uncertainty (e.g. confidence intervals)
☐	☒	For null hypothesis testing, the test statistic (e.g. *F*, *t*, *r*) with confidence intervals, effect sizes, degrees of freedom and *P* value noted<br>*Give P values as exact values whenever suitable.*
☐	☒	For Bayesian analysis, information on the choice of priors and Markov chain Monte Carlo settings
☒	☐	For hierarchical and complex designs, identification of the appropriate level for tests and full reporting of outcomes
☒	☐	Estimates of effect sizes (e.g. Cohen's *d*, Pearson's *r*), indicating how they were calculated

Our web collection on statistics for biologists contains articles on many of the points above.

Software and code

Policy information about availability of computer code

Data collection	All data extraction for this study was performed in Google Earth Engine, which provides the ability to compute LiDAR (GEDI) statistics and analyze the entire data records with high computational efficiency. Google Earth Engine is a cloud-based infrastructure that enables “access to high-performance computing resources for processing very large geospatial datasets”. It consists of “a multi-petabyte analysis-ready data catalogue co-located with a high-performance, intrinsically parallel computation service”.
Data analysis	Data analysis was performed on R (version 4.2.2).

For manuscripts utilizing custom algorithms or software that are central to the research but not yet described in published literature, software must be made available to editors and reviewers. We strongly encourage code deposition in a community repository (e.g. GitHub). See the Nature Portfolio guidelines for submitting code & software for further information.

Data

Policy information about availability of data

All manuscripts must include a data availability statement. This statement should provide the following information, where applicable:

- Accession codes, unique identifiers, or web links for publicly available datasets
- A description of any restrictions on data availability
- For clinical datasets or third party data, please ensure that the statement adheres to our policy

To ensure full reproducibility and transparency of our research, we provide all of the data analysed during the current study. The data are permanently and publicly available on a Github and Zenodo repository (immediately upon publication or for reviewers).

Research involving human participants, their data, or biological material

Policy information about studies with human participants or human data. See also policy information about sex, gender (identity/presentation), and sexual orientation and race, ethnicity and racism.

Reporting on sex and gender	*Use the terms sex (biological attribute) and gender (shaped by social and cultural circumstances) carefully in order to avoid confusing both terms. Indicate if findings apply to only one sex or gender; describe whether sex and gender were considered in study design; whether sex and/or gender was determined based on self-reporting or assigned and methods used. Provide in the source data disaggregated sex and gender data, where this information has been collected, and if consent has been obtained for sharing of individual-level data; provide overall numbers in this Reporting Summary. Please state if this information has not been collected. Report sex- and gender-based analyses where performed, justify reasons for lack of sex- and gender-based analysis.*
Reporting on race, ethnicity, or other socially relevant groupings	*Please specify the socially constructed or socially relevant categorization variable(s) used in your manuscript and explain why they were used. Please note that such variables should not be used as proxies for other socially constructed/relevant variables (for example, race or ethnicity should not be used as a proxy for socioeconomic status). Provide clear definitions of the relevant terms used, how they were provided (by the participants/respondents, the researchers, or third parties), and the method(s) used to classify people into the different categories (e.g. self-report, census or administrative data, social media data, etc.) Please provide details about how you controlled for confounding variables in your analyses.*
Population characteristics	*Describe the covariate-relevant population characteristics of the human research participants (e.g. age, genotypic information, past and current diagnosis and treatment categories). If you filled out the behavioural & social sciences study design questions and have nothing to add here, write "See above."*
Recruitment	*Describe how participants were recruited. Outline any potential self-selection bias or other biases that may be present and how these are likely to impact results.*
Ethics oversight	*Identify the organization(s) that approved the study protocol.*

Note that full information on the approval of the study protocol must also be provided in the manuscript.

Field-specific reporting

Please select the one below that is the best fit for your research. If you are not sure, read the appropriate sections before making your selection.

☐ Life sciences ☐ Behavioural & social sciences ☒ Ecological, evolutionary & environmental sciences

For a reference copy of the document with all sections, see nature.com/documents/nr-reporting-summary-flat.pdf

Ecological, evolutionary & environmental sciences study design

All studies must disclose on these points even when the disclosure is negative.

Study description	This study consists in analyzing the spatial and temporal effects of human-driven forest disturbances (including forest degradation due to selective logging, fire and edge effect and forest regrowth) on forest vertical canopy structure using around 39 million GEDI measurements across the pan-tropical belt.
Research sample	The research sample consists of LiDAR (GEDI) samples over different forest types from the JRC-TMF dataset (https://forobs.jrc.ec.europa.eu/TMF/). It covers intact (~23 million sample plots), degraded (~4.5 million), edge (~8.5 million) and regrowing (~1.1 million) tropical moist forests, representing large gradients of environmental, climatic and anthropogenic conditions of the humid tropics. Regarding edge effects, the sample covers forest at various distances to non-forest edge (i.e. urban or agricultural areas), varying from 60m to 10km. On the temporal aspects, the sample covers the last 33 years of forest disturbances and recovery (1990-2022) across various combination of disturbance types representative of the main direct drivers of forest degradation in the tropics (selective logging, fire and edge effect).

Sampling strategy	The sampling size depended on the availability of GEDI data (filtered to its highest quality). The resulting sampling sizes for each forest dynamics and for each types of forest disturbances are reported in the manuscript as numbers and maps.
Data collection	Data collection was based on the extraction of GEDI-derived canopy structure metrics (canopy height - RH98, height of median energy - RH50 and aboveground biomass density - AGBD) from 2019-2022 for different forest types captured by the JRC-TMF dataset. We created a fishnet grid of 1.5*1.5 degrees (~167km) covering the pantropical spatial domain. GEDI-derived metrics were extracted within each grid cell of this fishnet system. Ancillary datasets on selective logging (visual delineation of selective logging areas covering Brazil, French Guiana, Guyana, Cameroon, Central African Republic, Gabon, Congo, the Democratic Republic of Congo, Indonesia, Malaysia and Papua New Guinea) and forest fire (Global forest loss due to fire from the Global Land Analysis and Discovery) were used to refine the attribution of forest disturbance type.
Timing and spatial scale	The GEDI data was acquired from 2019 to end of 2022. It was analyzed on a yearly basis to ensure good precision in the assessment of the years since the last disturbance, the years since edge creation, or the age of forest regrowth. Forest cover dynamics of degradation, recovery and regrowth covered the period 1990-2022 coinciding with the monitoring period of forest cover change of the JRC-TMF dataset. Deforestation risk analysis was done on tropical moist forest cover change between 2020-2022 (1-3 years after the acquisition of GEDI canopy structure measurement). The spatial scale covers the tropical humid forest cover as defined by the Food and Agriculture Organization of the United Nations.
Data exclusions	GEDI data not passing the different quality filters (e.g. power beam mode, night acquisition, quality flags, slope less than 20 degrees, not classified as urban or water, beam sensitivity higher than 0.95) or forest definition (canopy height more than 5m) were excluded from the analysis. Mangrove forest were excluded from the analysis as periodical tidal floods will impact the consistent estimation of canopy height over time. Afforestation was excluded from the analysis due to the limited number of GEDI samples.
Reproducibility	All attempts to repeat the experiment were successful.
Randomization	Not relevant, as we used all the data to perform our analysis.
Blinding	Not relevant for this field (geoscience and forestry).

Did the study involve field work? ☐ Yes ☒ No

Reporting for specific materials, systems and methods

We require information from authors about some types of materials, experimental systems and methods used in many studies. Here, indicate whether each material, system or method listed is relevant to your study. If you are not sure if a list item applies to your research, read the appropriate section before selecting a response.

Materials & experimental systems

n/a	Involved in the study
☒	☐ Antibodies
☒	☐ Eukaryotic cell lines
☒	☐ Palaeontology and archaeology
☒	☐ Animals and other organisms
☒	☐ Clinical data
☒	☐ Dual use research of concern
☒	☐ Plants

Methods

n/a	Involved in the study
☒	☐ ChIP-seq
☒	☐ Flow cytometry
☒	☐ MRI-based neuroimaging

Plants

Seed stocks	*Report on the source of all seed stocks or other plant material used. If applicable, state the seed stock centre and catalogue number. If plant specimens were collected from the field, describe the collection location, date and sampling procedures.*
Novel plant genotypes	*Describe the methods by which all novel plant genotypes were produced. This includes those generated by transgenic approaches, gene editing, chemical/radiation-based mutagenesis and hybridization. For transgenic lines, describe the transformation method, the number of independent lines analyzed and the generation upon which experiments were performed. For gene-edited lines, describe the editor used, the endogenous sequence targeted for editing, the targeting guide RNA sequence (if applicable) and how the editor was applied.*
Authentication	*Describe any authentication procedures for each seed stock used or novel genotype generated. Describe any experiments used to assess the effect of a mutation and, where applicable, how potential secondary effects (e.g. second site T-DNA insertions, mosiacism, off-target gene editing) were examined.*
